# Smart MXene Quantum Dot-Based Nanosystems for Biomedical Applications

**DOI:** 10.3390/nano12071200

**Published:** 2022-04-03

**Authors:** Siavash Iravani, Rajender S. Varma

**Affiliations:** 1 Faculty of Pharmacy and Pharmaceutical Sciences, Isfahan University of Medical Sciences, Isfahan 81746-73461, Iran; 2 Regional Centre of Advanced Technologies and Materials, Czech Advanced Technology and Research Institute, Palacký University in Olomouc, Šlechtitelů 27, 783 71 Olomouc, Czech Republic

**Keywords:** MXenes, MXene quantum dots, biocompatibility, toxicity, smart nanosystems, biomedical applications

## Abstract

MXene quantum dots (QDs), with their unique structural, optical, magnetic, and electronic characteristics, are promising contenders for various pharmaceutical and biomedical appliances including biological sensing/imaging, cancer diagnosis/therapy, regenerative medicine, tissue engineering, delivery of drugs/genes, and analytical biochemistry. Although functionalized MXene QDs have demonstrated high biocompatibility, superb optical properties, and stability, several challenging issues pertaining to their long-term toxicity, histopathology, biodistribution, biodegradability, and photoluminescence properties are still awaiting systematic study (especially the move towards the practical and clinical phases from the pre-clinical/lab-scale discoveries). The up-scalable and optimized synthesis methods need to be developed not only for the MXene QD-based nanosystems but also for other smart platforms and hybrid nanocomposites encompassing MXenes with vast clinical and biomedical potentials. Enhancing the functionalization strategies, improvement of synthesis methods, cytotoxicity/biosafety evaluations, enriching the biomedical applications, and exploring additional MXene QDs are crucial aspects for developing the smart MXene QD-based nanosystems with improved features. Herein, recent developments concerning the biomedical applications of MXene QDs are underscored with emphasis on current trends and future prospects.

## 1. Introduction

The rise of MXenes and their (nano)structures with fascinating physical and chemical features such as photo-thermal stability, hydrophilicity, multimodal sensing/imaging capacities, large surface area, and thermal/electrical conductivity has astounded researchers worldwide [1,2,3]. Two-dimensional materials such as black phosphorus nanosheets, MXenes, graphene, and molybdenum disulfide with their remarkable structures and features as well as diverse applications and synthesis processes have attracted the attention of many researchers, in particular for developing high-efficiency and specific hybrids or assemblies [4,5]. For instance, nanosheets of black phosphorus exhibited the advantages of intrinsic photoacoustic properties and suitable biocompatibility/biodegradability, which make these materials promising candidates for a variety of biomedical explorations in the field of cancer theranostics, photothermal/photodynamic therapy, drug/gene delivery, and tissue engineering [6,7,8,9]. Despite all these valuable properties, their intrinsic instability and the associated degradation is one of the important challenges for large scale production, thus restricting their applicability [6,7,10]. However, the fascinating optical characteristics of MXene quantum dots (QDs) such as light absorption, photoluminescence, and electrochemiluminescence make them alluring candidates for appliances in biomedicine, optoelectronic catalysis, and optoelectronic devices. Additionally, the significant electronic features of MXene QDs should be further evaluated. In particular, their magnetic properties have rarely been illustrated by experimental studies, and bare and terminated MXenes with magnetism were introduced by density-functional theory calculations [11]. In comparison to the other reported QDs based on graphene, carbon, graphitic carbon nitride (g-C_3_N_4_), and black phosphorus, MXene QDs can be considered as promising catalysts and co-catalysts, as they display some attractive benefits namely the ease of surface functionalization deploying –O, –OH, and –F groups, high electro-conductibility, controllable band structure, low toxicity/biocompatibility, exclusive photochemical robustness, tunable optical features, and strong catalytic potentials [12,13]. Several reviews have focused on graphene, g-C_3_N_4_, and other QDs focusing on their potential biomedical appliances [14,15]. Herein, we comprehensively discussed about MXene QD-based nanosystems as smart platforms with biomedical potentials. The present manuscript may be of interest to a broad readership and nanoscientists in the fields of nanomedicine, nanotechnology, MXene QDs, cancer theranostics, advanced nanomaterials, biomaterials, as well as in emerging nanotechnology processes and technologies. However, still more elaborative studies should be focused on clinical translation; systematic studies are anticipated for long-term toxicity, histopathology, biodistribution, biodegradability, and photoluminescence properties. The MXene QDs with unique chemical and physical properties will hopefully find their distinctive position in near future on the research platform focusing in the bio- and nanomedicine arena. As an example, Ti_3_C_2_T_x_ MXene-derived QDs with abundant active sites were introduced as N_2_ reduction reaction electrocatalysts with high efficiency [16]. Density functional theory calculations demonstrated that surface functional groups (especially, hydroxyl groups) played a crucial role in their electrocatalytic activity. Hydroxyl-rich MXene QDs introduced as N_2_ reduction reaction catalysts via alkalization and intercalation process could provide an NH_3_ yield and Faradaic efficiency of 62.94 µg h^−1^ mg^−1^ _cat_ and 13.30% at −0.50 V, respectively, after the optimization process [16]. 

Currently, several innovative synthetic strategies are being pursued for various types of smart nanosystems and nanostructures encompassing MXenes, including Ti_2_CT_x_, Ti_3_C_2_T_x_, Ta_4_C_3_T_x_, Nb_2_CT_x_, among others [17,18,19,20]. MXenes have been prepared by chemical vapor deposition [21], electrochemical synthesis [22], hydrothermal/solvothermal synthesis [23], urea glass technique [24], ultrasonication [25], microwave-assisted synthesis [26], and various etching techniques such as electrochemical-, alkali-, molten salt- and in situ hydrofluoric acid-forming etching routes (Table 1) [27,28,29]. For instance, MXene (Ti_3_C_2_) QDs were synthesized using a facile hydrothermal technique which exhibited excitation-dependent photoluminescence spectra with quantum yields of up to ≈10% due to strong quantum confinement [30]. Additionally, biocompatible MXene QDs were fabricated using an effective fluorine-free technique as nano-agent for photothermal therapy purposes with high efficiency and no noticeable toxicity. This synthesis technique demonstrated higher safety, simplicity, and environmentally benign advantages when compared with the traditional methods, which utilized hazardous and time-consuming procedure of hydrofluoric pretreatment [31]. Bai et al. [32] synthesized the green fluorescence nitrogen, phosphorus-doped Ti_3_C_2_ MXene QDs through a facile microwave-assisted technique, with an advantage of reduction in reaction time; ensued QDs endowed with suitable dispersibility illustrated excitation-dependent photoluminescence and anti-photobleaching features [32]. Gogotsi and co-workers have critically discussed the guiding principles and the essential precautions for reducing the risk of hazardousness and environmental toxicity during the synthesis of MXenes; methods with high safety, reproducibility, and reliability should therefore be the main objective [33]. MXenes have been widely explored as attractive inorganic two-dimensional candidates for assorted applications in gene/drug delivery, imaging or sensing, tissue engineering, regenerative medicine, and cancer theranostics [5,34,35,36]. Remarkably, their surfaces can be suitably functionalized or modified to enhance biocompatibility, functionality, selectivity/sensitivity, and smart targeting features thus rendering them attractive candidates for biomedical and pharmaceutical applications [37,38,39]. However, it appears that further explorations are needed regarding the biocompatibility, genotoxicity, and cytotoxicity of MXene-based materials or MXenes themselves, in vitro and in vivo [40]. The association of cytotoxicity of MXenes with the size/morphology, exposure time, functional groups, oxidative state, synthesis techniques, and type/dose administration have been reported, the size of MXenes being a vital parameter for their internalization by the targeted cells via endocytosis [40]. 

The dimensional reduction of MXenes from two-dimension to zero-dimension QDs can provide exceptionally unique characteristics and functionalities [52]. However, this field of science is still in its infancy as more elaborative studies are essential for analyzing and adjusting their biocompatibility, biodegradability, long-term toxicity/cytotoxicity, histopathology, fluorescence emission, pH- and photo-stability, and other important criteria [53,54]. Although several reviews have been appeared on the synthesis, applications, and properties of MXenes and their nanocomposites [53,55,56,57,58,59], but the necessity of a perspective article around the biomedical potentials of MXene QDs remains a void. Even though extensive and comprehensive research is still awaited on MXene QDs, these materials with their advantages of good biocompatibility, high photoluminescence features, remarkable selectivity/sensitivity to targets, abundant active catalytic sites, significant electrical conductivity, easily tunable structures, fascinating optical properties, suitable dispersibility, biodegradability, and low toxicity, will soon find their distinctive position on the research platform focusing on the biomedicine and nanomedicine arena. These nanomaterials can be applied for developing next-generation of smart nanosystems with clinical and biomedical applicability [5,60,61,62]. Herein, recent advances relating to the appliance of MXene QDs in biological sensing/imaging, cancer therapy/diagnosis, tissue engineering, regenerative medicine, and antioxidants have been deliberated, with emphasis on imperative challenges and imminent outlooks. 

## 2. Biomedical Advancements

### 2.1. Biological Sensing and Imaging 

Conventional fluorescent probes may have handicap of non-biodegradability, long-term biological toxicity, and unstable and inadequate fluorescence signal output. Thus, creatively-designed fluorescent agents with significant biodegradability and photostability should be further explored. In one study, Ti_3_C_2_ MXene QDs with high photoluminescence properties were prepared via a simple hydrothermal technique and were deployed as zinc ion sensors and biocompatible multicolored cellular imaging probes [30]. Furthermore, the prepared Nb_2_C QDs (with quantum yield of up to 19%) demonstrated green fluorescence and high pH- and photo-stability; the S, and N doping on Nb_2_C improved the quantum yield of the Nb_2_C dots. This fluorescent probe is a highly efficient candidate for three-dimensional (3D) brain organoid labeling, which can be applied in biological sensing and labeling [63]. Li et al. [64] reported the elevated photoluminescence quantum yield up to 18.7% for *N*-doped Ti_3_C_2_, providing a suitable platform for the sensitive detection of Fe^3+^ with the detection perimeter of up to 100 μM. The smart QDs with high sensitivity are suitable candidates for sizeable biosensing applications [64], the crucial parameter in labeling being the toxicity of the ensued MXene QDs. In one study, Ti_3_C_2_ and Nb_2_C MXene QDs were evaluated for their possible toxicity to the human umbilical vein endothelial cells (in vitro). Consequently, Ti_3_C_2_ QDs (up to 100 μg mL^−1^) could induce significant cytotoxicity after 24 h by promoting the autophagic dysfunction; both the QDs had internalization and could promote the release of IL-8 and interleukin 6 (IL-6). Further, Ti_3_C_2_ QDs could enhance the ratio of LC3B-II/I, biomarkers of autophagy, autophagic substance p62, and beclin-1 proteins; pro-caspase 3 was efficiently stimulated by Ti_3_C_2_ QDs. Notably, Nb_2_C QDs exhibited better biocompatibility with the examined cells than Ti_3_C_2_ QDs, revealing the important functions of the composition and structure of MXenes in ensued toxicity [65]. 

As has been specified in the documented literature, one of the crucial challenging issues is the biodegradability aspects of the synthesized nanomaterials, affecting their long-term toxicity, adverse reactions, and their excretion from the body after fulfilling the purposed functions [58,66,67]. Nb_2_C MXene QDs with high photo- and chemical-stable fluorescence emission was fabricated via physicochemical exfoliation in tetrapropylammonium hydroxide deploying ultrasonic irradiation. These QDs with good biocompatibility and biodegradability illustrated their great potentials in fluorescence imaging and sensing of heavy metal ions (e.g., Fe^3+^) [25]. Besides, fluorescent MXene (Ti_3_C_2_) QDs functionalized with protein bovine serum albumin were synthesized by hydrothermal technique. These quasi-spherical QDs (~2 nm) exhibited unique photo-physical attributes with higher stability at different physiological conditions. In the presence of Fe^3+^ ions, the fluorescence intensity of these QDs was selectively quenched; ensued probes from these QDs illustrated good selectivity and sensitivity towards Fe^3+^ ions, providing practical potentials to generate sensitive sensors endowed with biocompatibility features [68].

Ti_3_C_2_ MXene QDs were fabricated with strong two-photon white fluorescence and high stability of white emission. Hybrid nanocomposites were prepared via the polymerization of these QDs in polydimethylsiloxane solution, which can be applied in different fields of optics and imaging [69]. Notably, the environmentally-benign synthesis of smart MXene QDs has been initiated by researchers to prevent the deployment of complicated instruments and hazardous materials as well as high-cost tactics requiring high energy and pressure. Dhingra et al. [70] fabricated biocompatible zero-dimensional Ti_3_C_2_T_x_ MXene QDs with the cellular uptake ability for biomedical purposes; the engineered nanostructures have enough transferring potentials from vascular endothelial cells as the obstruction between organs and blood. It was indicated that these QDs could be extemporaneously uptaken into human endothelial cells within 24 h of cell culture. They were localized with high stability, no noticeable modifications in cell morphology, and robust auto-fluorescence features at different emission-excitation wavelengths allowing the post-transport examination and tracking [70]. 

MXene QDs have been explored as attractive fluorescent probes for various photoelectric conversion, optical sensing, and biological imaging appliances [50]. Xu et al. [50] reported the hydrothermally synthesized nitrogen (N)- and phosphorus (P)-functionalized photoluminescent Ti_3_C_2_ MXene QDs with high photo- and pH- constancy, which were further studied for macrophage labeling as fluorescent probes. Furthermore, these QDs could function as inexpensive, eco-friendly, and label-free fluorescence system with high sensitivity for the detection of Cu^2+^ [50]. Additionally, *N*-doped Ti_3_C_2_ MXene QDs with ~1 nm depth and ~6.2 nm size was synthesized via amine-assisted solvothermal tailoring technique. These QDs displayed various fluorescence-quenching reactions to different metallic cations, providing sensitive revelation opportunities for Cu^2+^ [71]. Besides, a composite film endowed with the potential of photo-electrochemical biosensing applicability was designed from titanium dioxide (TiO_2_) inverse opal photonic crystals and Ti_3_C_2_ MXene QDs [42]. As a result, the electrode prepared from this composite illustrated significant stability and sensitivity/selectivity for detecting glutathione in buffered solution and cell extracts, offering promising potentials for early precaution and diagnosis of diseases [42].

MXene-based QDs illustrated great potentials for enhancing the specificity and sensitivity of sensors deployed for detecting proteins, genes, and viral particles [11]. For instance, to identify the histidine in human serum, amino-functionalized Ti_3_C_2_ MXene QDs with bright blue fluorescence and high bio-affinity were prepared [72]. Furthermore, biocompatible aerogels with good stability have been constructed from MXene QDs and watermelon peel by immersing freeze-dried fresh watermelon peel into the QD dispersion; these nanosystems demonstrated suitable functionality in biosensing appliances [73]. Chen et al. [74] functionalized Nb_2_C MXene QDs with octadecanethiol for the particular recognition of N-gene of SARS-CoV-2 using a tag-free surface plasmon resonance (SPR) aptasensor; these QDs could improve the bio-affinity toward aptamer and enhance the SPR response. The conformation of immobilized aptamer strands was altered for specific binding with N-gene, after the existence of SARS-CoV-2 N-gene. Also, the distance was enlarged among the aptamer and the SPR gold chip altered with Nb_2_C-SH QDs through covalent attachment of the Au-S bond, providing an alteration in the laser irradiated surface plasmon resonance signal (the wavelength = 633 nm). The low limit of detection (LOD) for the constructed QD-based aptasensor was ~4.9 pg mL^−1^ for the N-gene (dilution was between 0.05 to 100 ng mL^−1^), enabling immense practical applications for qualitatively analyzing N-gene from various samples [74]. 

**Figure 1:**
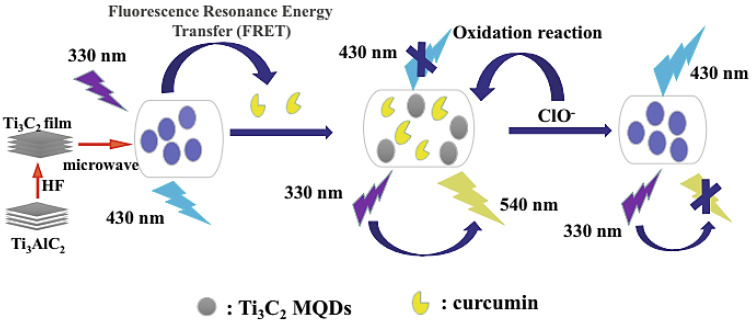
Ti_3_C_2_ MXene QDs applied for designing a sensing platform to specifically detect ClO^−^ and curcumin. Adapted with permission from Ref. [26]. Copyright 2021 Springer Nature.

N, boron (B)-Ti_3_C_2_ MXene QDs were prepared for designing a ratiometric fluorescence sensing system for point-of-care detection of tetracycline antibiotic (LOD = 20 nM). These QDs exhibited suitable stability, optical features, and water solubility. In the presence of tetracycline, blue fluorescence emission of the prepared MXene QDs was reduced while the emission of red fluorescence of Eu^3+^ was slowly increased [75]. Liu et al. [26] innovatively designed smart system from Ti_3_C_2_ QDs for detecting hypochlorite (ClO^−^) and curcumin. ClO^−^ could oxidize the methoxy and phenolic groups of curcumin to quinones, and the re-establishment of the fluorescence of MXene QDs was reported. For curcumin, the linear uncovering range was ~0.05–10 μM with LOD being ~20 nM. Furthermore, the linear detection limits for ClO^−^ were ~25–150 μM and 150–275 μM, with LOD of ~5 μM (Figure 1) [26]. 

### 2.2. Tissue Engineering and Regenerative Medicine 

Ti_3_C_2_ MXene QDs have been explored for their potential immunomodulatory effects with the express purpose of increasing material-based tissue restoration following injury [41]. As a result, these QDs had innate immunomodulatory attributes and specifically lessened the activation of human CD4^+^IFN-γ^+^ T-lymphocytes at the same time invigorating the growth of immunosuppressive CD4^+^CD25^+^FoxP3^+^ regulatory T-cells in an aroused lymphocyte populace. Additionally, they had good biocompatibility with -derived mesenchymal stem cells derived from bone marrow and fibroblasts derived from stimulated pluripotent stem cells. Notably, Ti_3_C_2_ QDs were integrated into a chitosan-centered hydrogel to produce a three-dimensional system with improved physicochemical features for the delivery of stem cells and healing of tissues. The prepared hydrogel composites illustrated improved conductivity and at the same time maintained thermo-sensitivity and injectability. These kinds of smart biomaterials can assist in bridging the translational gap for materials, repairing the tissue on the basis of stem cell-based therapeutic strategies, and treating the inflammatory/degenerative disorders [41]. In another study, the immuno-engineered Ta_4_C_3_T_x_ MXene QDs were developed for treating transplant vasculopathy, in vivo. These smart QDs with interesting antiapoptotic and anti-inflammatory features can be considered for biomedical engineering. Remarkably, Ta_4_C_3_T_x_ QDs were impulsively uptaken into antigen-presenting endothelial cells and could modify the expression of surface receptor to diminish their activation of allogeneic T-lymphocytes; the cellular/structural alterations of early allograft vasculopathy were ameliorated [76]. 

### 2.3. Cancer Theranostics

**Figure 2:**
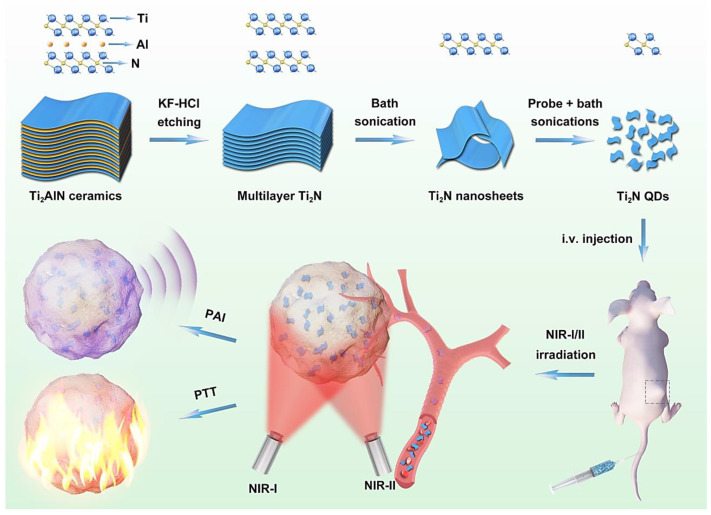
The biodegradable Ti_2_N QDs designed for photoacoustic imaging (PAI)-guided photothermal therapy (PTT) of cancers in both near-infrared (NIR)-I/II bio-windows, after intravenous (I.V) injection. Adapted with permission from Ref. [82]. Copyright 2020 Elsevier.

Various fluorescent nanoparticles and QDs have been widely explored for cancer theranostics. However, several challenges regarding the surface functionalization, biocompatibility, simplicity of synthesis process, and possible environmental hazards have impeded their future clinical and biomedical appliances [77]. Therefore, the investigational priority should be focused on one-step greener synthesis techniques with cost-effectiveness, high yields/safety, simplicity, and optimized conditions to generate multifunctional nanostructures with high biocompatibility and efficiency. The hybridization and surface modification using bioactive functional agents are good examples for enhancing the properties of the prepared QDs that prevent degradation and improve their stability, reusability, targeting/specificity, and functionality [78,79,80]. Additionally, nano-scale catalysts based on Fenton or Fenton-like reactions have been developed to amplify the intracellular oxidative stress for specific tumor/cancer therapy. The crucial challenges are the low efficacy of catalysts, possible toxicity, and poor biocompatibility [81]. In one study, non-oxidized Ti_3_C_2_T_x_ MXene QDs with inhibitory effects on cancerous cells (the suppression rate was ~91.9%) and high biocompatibility were fabricated via a self-planned micro-outburst approach. Mechanistically, the Ti^3+^ from these QDs upon reaction with hydrogen peroxide (H_2_O_2_) in the tumor microenvironment could efficiently generate extremely toxic hydroxyl radicals to increase the tumor microvascular penetrability for synergistically killing the cancerous cells [81]. Additionally, Ti_2_N QDs (~5 nm) fabricated by a top-down strategy exhibited suitable biodegradability and biocompatibility features, providing photothermal therapy of cancers with more efficacy than the routine inorganic photothermal materials with poor biodegradability disadvantage. These QDs retained good stability in their structures in the early stage of circulation in the body for imaging/therapeutic purposes while aggregating in the targeted cancerous sites after 4 h of injection, and could be easily cleared from the body after the usage. Such MXene QD-based nanosystems enabled photoacoustic imaging-guided photothermal therapy of cancers in both NIR-I/II bio-windows with high biosafety (Figure 2) [82]. 

**Figure 3:**
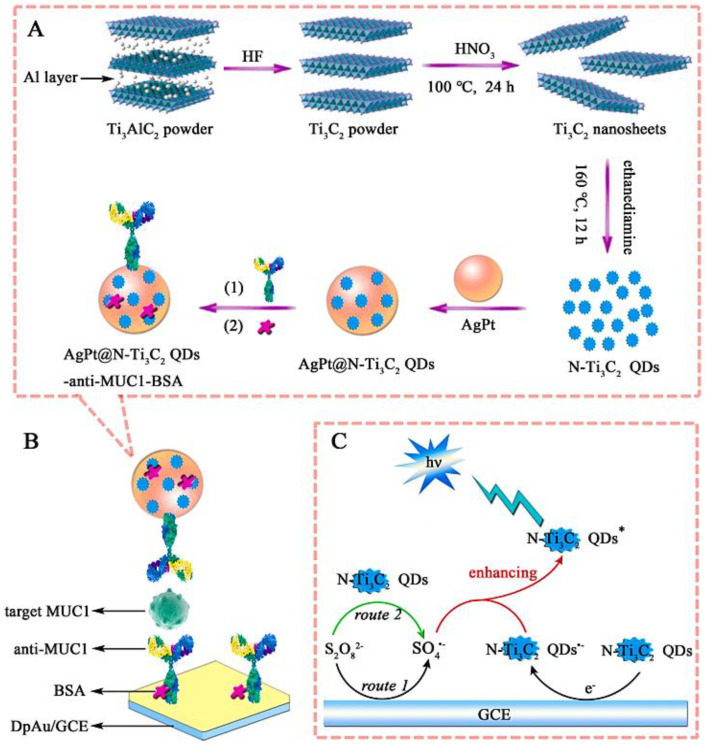
The preparation of AgPt@N-Ti_3_C_2_ MXene QDs-anti-MUC1-BSA bio-conjugates (**A**), for designing the electrochemiluminescence (ECL) immunosensor (**B**), and the related ECL mechanism of NTi_3_C_2_ QDs/S_2_O_8_^2−^ system (**C**). BSA, bovine serum albumin; HF, hydrofluoric acid. Adapted with permission from Ref. [83]. Copyright 2022 Elsevier.

To design biocompatible nano-agents for simultaneous photoacoustic imaging and photothermal therapy of cancers and tumors, the innovative synthesis methods are sought in which the hazardous and time-consuming procedures are avoided. Liu et al. [31] introduced a fluorine-free technique with safety and simplicity in which the abundant Al oxoanions were altered on the surfaces of MXene QDs via this methodology. These QDs exhibited robust and wide-ranging absorption in the NIR region, with a photothermal conversion efficiency of ~52.2% and an extinction coefficient of 52.8 Lg^−1^ cm^−1^ at 808 nm [31]. Additionally, N-Ti_3_C_2_ MXene QDs with high chemical stability, metal conductivity, electrochemiluminescence efficiency and non-toxicity have been fabricated via a simple hydrothermal technique using ethylenediamine as the N source and Ti_3_C_2_ as the precursor (Figure 3). The electrochemiluminescent QDs were studied for designing a sensitive immunosensor to determine mucin 1 (MUC1) that is related to the malignancy development (LOD = 0.31 fg mL^−1^) [83].

### 2.4. Antioxidant Effects

**Figure 4:**
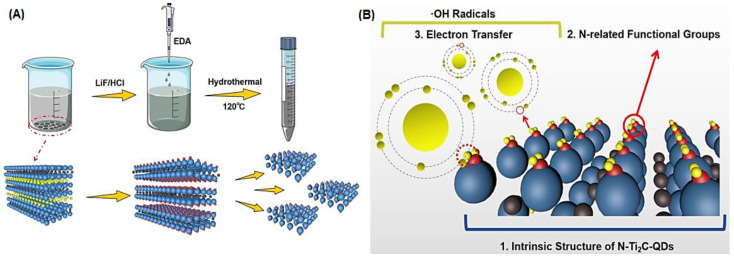
(**A**) The fabrication process of N doped Ti_2_C MXene QDs, and (**B**) their antioxidant activity. Adapted with permission from Ref. [47]. Copyright 2021 American Chemical Society.

MXene QDs exhibited suitable antioxidant properties, but they are typically oxidized during hydrothermal fabrication and some defects can occur on their constructions that reduce antioxidant capabilities [47]. In one study, Ti_2_C MXene QDs were protected from damages during production by applying ethylenediamine as a precursor to introduce N element. The prepared *N*-doped Ti_2_C MXene QDs had strong antioxidant activity as superb scavenger of radicals (•OH), protector of dyes, and reducer of KMnO_4_ (Figure 4) [47]. Qu et al. [84] have investigated the reactive oxygen species (ROS) scavenging and antioxidant capabilities of *N*-doped Ti_3_C_2_ QDs and their attendant mechanism. Consequently, the electrochemical interaction between the prepared QDs and free radicals was promoted by doping with the N element, which enhanced the antioxidant activities. In addition, the hydroxyl radical quenching procedure was revealed by density functional theory simulations, confirming the stimulatory effects of doped N element on the ability of free-radical absorption, especially for functional groups encompassing –F and –O in the QDs. The ensued *N*-doped Ti_3_C_2_ QDs were highly sensitive to rapidly detect H_2_O_2_ (between the 5 nM and 5.5 μM ranges), and the related LOD was ~1.2 nM within 15 s, which demonstrated sensitive and real-time H_2_O_2_ sensing capacity of this QD-based system [84].

**Figure 5:**
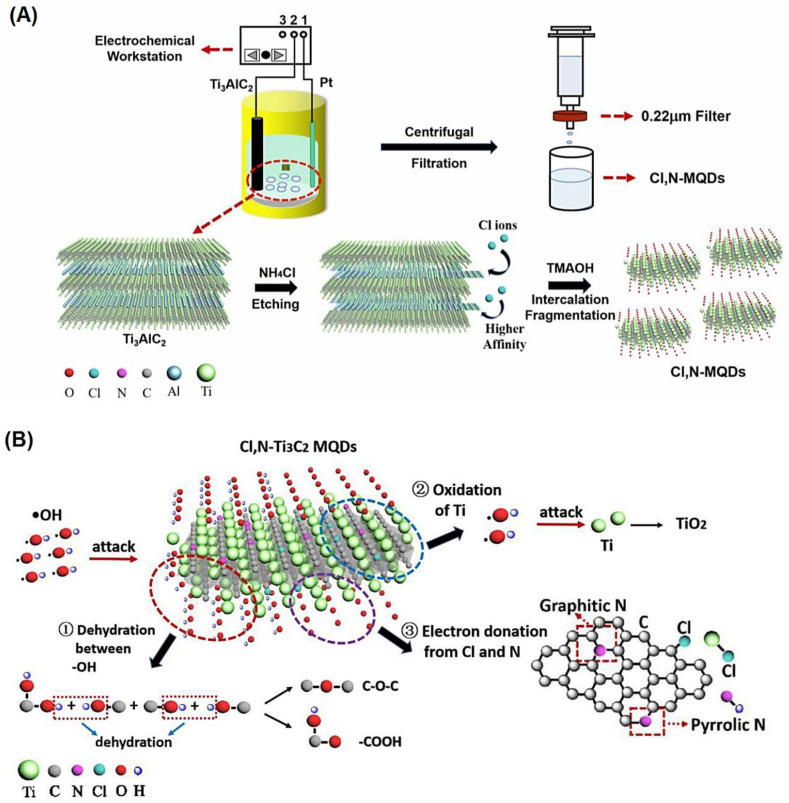
(**A**) The fabrication process of Cl and N co-doped Ti_3_C_2_ MXene QDs, and (**B**) their scavenging mechanism towards •OH radicals. MQDs, MXene QDs. Adapted with permission from Ref. [85]. Copyright 2021 Elsevier.

Chlorine (Cl) and N co-doped Ti_3_C_2_ MXene QDs have been prepared with large surface-to-volume ratio and high radical scavenging action (~93.3%). The QDs (~3.45 nm) were directly stripped from bulk Ti_3_AlC_2_ via an energy-saving and green electrochemical etching process, while N and Cl were presented to carbon frame and titanium peripheries in the engraving procedure by electrochemical reactions between selected electrolytes and Ti_3_C_2_ skeleton, respectively (Figure 5) [85]. Qi et al. [86] reported the high scavenging activity of Ti_3_C_2_T_x_ MXene against 2,2-diphenyl-1-picrylhydrazyl (95% in 10 min) at a low dose (0.06 mg mL^−1^). Furthermore, non-enzymatic antioxidant tactics based on MXene-based materials was illustrated for the treatment of diseases [87]. It was indicated that nanosheets of Ti_3_C_2_-polyvinylpyrrolidone demonstrated high biocompatibility and biodegradability as well as superb chemical reactivity toward multiple ROS for treating acute kidney injury [87]. Several studies have been undertaken on antioxidant and scavenging properties of MXenes and MXene-based nanocomposites. However, comprehensive investigations on underlying mechanisms of these properties are still necessary, especially in the treatment of ROS-related diseases.

## 3. Challenges and Future Perspectives

MXenes and their derivatives have offered promising properties for diverse theranostics and photo-medicine purposes due to their fascinating electrical conductivity, high stability, robust nonlinear optical response, and tunable terminated surface [60]. Although MXene QDs have garnered diverse attention in bio- and nanomedicine, several challenges pertaining to their possible applications for cancer theranostics are still awaiting resolution, especially in terms of their biocompatibility, toxicity, and specificity. Clinical trials and systematic in vivo/in vitro studies are also anticipated for examination of their long-term toxicity, histopathology, biodistribution/biodegradability, and photoluminescence properties. On the other hand, it appears that their biological sensing and imaging applications have been restricted by their non-specific adsorption (although, in some cases, the improvement in properties such as biodegradability and photostability of MXene QDs has been achieved). As discussed earlier, Nb_2_C QDs prepared by optimized ultrasound-assisted technique had significant on photo-chemical stability, higher biocompatibility, and better biodegradability properties, providing fluorescence imaging and sensing [25]. Although MXene QDs have displayed excellent photothermal conversion, fluorescence behavior, and photonic/photo-electronic features, important challenging issues involving the clarification of the electron structure-related mechanisms for improving the properties of MXene QDs and extending these properties to newer fields of explorations should be considered. Although several functionalization strategies such as surface modification, heteroatom doping, and fabricating composites have been reported, but more elaborative studies are still required, especially for improving the optical, mechanical, electronic, and magnetic properties of MXene QD-based nanosystems [12,16,46,48,51].

To employ MXene QDs in tissue engineering and regenerative medicine, there is still a need for more extensive and comprehensive research so that these materials can find their place as a suitable adjutant or platform. It has been emphasized that when MXene QDs were incorporated in the form of (nano)composites (e.g., chitosan-based hydrogels), innovative platforms could be obtained with improved thermo-sensitivity, conductivity, and injectability, helping to bridge the translational gaps in materials and stem cell-based therapeutic tactics for repairing tissues and treating degenerative/inflammatory ailments [41]. Besides, MXenes nanomaterials may generate the intracellular oxidative stress for cancer theranostics, and biocompatibility around their radical activity is another lingering question [88,89]. Given that these materials produce the intracellular oxidative stress, it is difficult for these materials to be deployed as antioxidants. Consequently, precise analyses of their antioxidant mechanisms and means to improve the performance of these compounds should be considered. In terms of subcellular nanomedicine appliances, it was reported that MXene QDs could be efficiently and impulsively internalized into human vascular endothelial cells, with no need of any uptake enhancing tactics [70].

The routine methods for synthesizing MXenes and their derivatives may contain the environmentally unsafe/toxic etchants (e.g., hydrofluoric acid or zinc chloride) and organic solvents (for the delamination/intercalation) that need to be replaced by environmentally-benign processes and eco-friendly materials. There is a vital need for fluorine-free and greener synthesis methods with sufficient flexibility for up-scalable production in higher yield. Theoretically designed MXenes should eventually be sent to the laboratory for extensive analysis. The deployment of the hydrofluoric acid etching method may generate MXenes surface terminal groups (such as –OH, –F, and –O) that appear randomly or non-uniformly on their surfaces [53,55,56,57,58,59,90]. Thus, further evaluations are required for their surface modification or functionalization. Recent advances in optimal changes and surface functionalization of MXene-based nanostructures have also been discussed in detail [66]. It appears that due to the existing defects and difficulty in surface functionalization and control of size/morphology of MXenes in top-down synthesis strategies, investigations should be broadly geared towards a more coherent bottom-up methods with optimized and mild conditions. Additionally, the quantum yields and photoluminescence of the obtained MXenes are typically low in the UV region of the spectrum, thus restricting their bio-imaging, biomedical diagnosis, and fluorescence applications. This indicates a need to produce MXene QDs with optimal properties and high quantum yield which can pave the way for a broad range of applications in theranostics, imaging technology, immunoassays, among others [42,50,69]. As the most examined MXenes is Ti_3_C_2_T_X_, additional kinds of MXenes should be fabricated and evaluated for their possible biomedical potentials, focusing on their biocompatibility, long-term stability, and toxicity assessments. Finding the suitable means (e.g., the hybridization) and modification methods for reducing the toxicity and controlling the morphology of MXene nanomaterials should be given higher priority in research for developing the practical value of these materials for manufacturing smart nanosystems [30,91,92,93,94,95].

## 4. Conclusions and Future Outlooks

Smart and multifunctional MXene QD-based nanosystems have shown superb photoelectric properties and compatibility, making them promising nanostructures for cancer theranostics, tissue engineering, drug delivery, biological sensing/imaging, antioxidants, regenerative medicine, and wound healing purposes. Despite extensive research on MXenes, they have not been comprehensively evaluated for their untapped biomedical potentials. In this context, MXene QDs with high quantum yield and photoluminescence properties should be further explored in immunoassays, fluorescence imaging technology, and biosensing purposes. It appears that due to the unique physicochemical properties of MXene QDs, special triumphs can be realized in the fabrication and design of smart nanosystems with clinical and biomedical potentials; the selective and sensitive detection of diverse molecules can be achieved by the MXene QD-based nanosystems. However, to move towards clinical applications, critical issues pertaining to their photo-chemical stability, long-term cytotoxicity/biosafety, biocompatibility, biodegradability, and controllable fluorescence sensing/imaging properties should be analytically assessed.

The biosafety and bio-functionality as well as selectivity/sensitivity and stability of MXene QD-based nanosystems can be improved by appropriate surface functionalization or modification of existing functional groups. However, the functionalization of MXene QDs is in its infant stage and further studies are recommended. Also, their possible adverse effects, cytotoxicity, and immune reactions are crucial parameters which should be considered analytically and clinically. Furthermore, the association of these factors in terms of their size, morphology, structure, and composition of MXenes needs more extensive and methodological investigations. The synthesis of MXene QDs via bottom-up or top-down methods with enough simplicity, low toxic or hazardous agents/chemicals, low temperature/pressure, mild reaction conditions, good dispersity/crystallinity, and large scale potentials still need to be given higher precedence. Routinely deployed techniques for the synthesis of MXenes involve the utilization of hazardous and environmentally unsafe agents such as hydrofluoric acid. In addition, some organic solvents have been utilized for intercalation and delamination processes, which can be toxic and damaging for the environment. Additionally, the conventional fabrication techniques do not have enough flexibility for large scale production, thus restricting their commercialization aspects. The application of etchants (e.g., zinc chloride or HCl/LiF) have not delivered high yield of production, except after optimization of reaction conditions; the synthesis process often suffers from complex and laborious steps. Future explorations should be focused on environmentally friendly, fluorine-free, simple, and cost-effective production of MXenes with higher yields. Advanced optimization and functionalization processes can also help in improving the functionality, stability, and biosafety criteria of MXene QDs. Many of the introduced MXene structures are designed theoretically and only a handful of them have been obtained on the laboratory scales. Thus, it is anticipated that with extensive efforts to optimize the synthesis and specific properties of these high-value compounds, a move towards industrialization, large scale production, and clinical usage can be realized, especially for rapid and intelligent diagnosis and treatment of diseases and tissue engineering and restoration.

## Figures and Tables

**Table 1:** Some notable examples of MXene QDs with biomedical applicability.

MXene QDs	Synthesis Methods	Applications	Ref.
Ti_3_C_2_	Hydrothermal synthesis	Immunomodulation	[41]
Ti_3_C_2_	Hydrothermal synthesis	Glutathione detection and photoelectrochemical biosensing	[42]
Ti_3_C_2_	Intercalation-ultrasound sysnthesis	Prostate-specific antigen detection	[43]
Mo_2_C	Ultrasound-assisted synthesis	(Bio)imaging and photothermal therapy	[44]
Mo_2_C	Molten salt (molybdenum acetylacetonate, NaCl, 800 °C for 2 h)	Nitrogen reduction reaction	[12]
V_2_C	Hydrothermal synthesis	(Bio)imaging, photothermal therapy, and tumor destruction	[45]
Ti_3_C_2_	Hydrothermal synthesis	Multicolor cellular imaging and Zn^2+^ detection	[30]
Ti_3_C_2_	Ultrasound-assisted synthesis; fluorine-free preparation	(Bio)imaging and photothermal therapy	[31]
Ti_3_C_2_	Hydrothermal synthesis	(Bio)imaging and pH sensor	[13]
Ti_3_C_2_	Hydrothermal synthesis	Enzyme assay and cell identification	[46]
Ti_2_C	Hydrothermal synthesis	Antioxidant effects	[47]
Ti_3_C_2_	Hydrothermal synthesis	Cytochrome c and trypsin detection	[48]
Ti_3_C_2_	Reflux technique	Glutathione detection	[49]
Ti_3_C_2_	Hydrothermal synthesis	Bioimaging, macrophage labeling, and Cu^2+^ detection	[50]
MoS_2_	Hydrothermal synthesis	Methanol oxidation reaction and oxygen reduction reaction	[51]
Ti_3_C_2_	Microwave-assisted technique	Detection of curcumin and hypochlorite (ClO^−^)	[26]

## Data Availability

Not applicable.
